# Scheduled Analgesic Regimen Improves Rehabilitation After Hip Fracture Surgery

**DOI:** 10.1007/s11999-013-2927-5

**Published:** 2013-03-30

**Authors:** Raymond Ping-Hong Chin, Chin-Hung Ho, Lydia Po-Chee Cheung

**Affiliations:** 1 Department of Orthopaedics and Traumatology, Queen Elizabeth Hospital, Hospital Authority, 3/Floor, M Block, 30 Gascoigne Road, Jordon, Kowloon, Hong Kong, China; 2 Orthopaedic Rehabilitation Centre, Kowloon Hospital, Hospital Authority, Hong Kong, China

## Abstract

**Background:**

Postoperative pain often is the limiting factor in the rehabilitation of patients after hip fracture surgery.

**Questions/purposes:**

We compared an approach using scheduled analgesic dosing with as-needed analgesic dosing in patients after hip fracture surgery, to compare these approaches in terms of (1) resting and dynamic pain intensity, (2) postoperative patient mobility, and (3) functional end points.

**Methods:**

We conducted a prospective cohort study of 400 patients who underwent surgical treatment of hip fractures at our hospital. The groups were formed sequentially, such that the first 200 patients formed the intervention group (treated with scheduled analgesic intake for the first 3 weeks after surgery), and the next 200 patients were the control group (treated using a protocol of analgesic administration on request). Resting and dynamic pain intensity, mobility, and functional performance were compared between the two analgesic protocols.

**Results:**

As expected, analgesic consumption was lower in the control group (tramadol doses, 27 versus 63; paracetamol doses, 29 versus 63). Despite the large difference in the amounts of analgesics consumed, resting and dynamic pain intensity showed improvement in each group and there was no difference between groups in terms of postoperative pain. However, there was a positive correlation between functional outcomes and analgesic consumption in the control group. The intervention group achieved higher functional performance on discharge (elderly mobility scale, 11 versus 8; functional independence measure, 88 versus 79). On discharge, fewer patients in the intervention group were wheelchair ambulators (3 versus 32), meaning more patients in the intervention group were able to walk.

**Conclusions:**

The study showed that a scheduled analgesic intake can improve the functional outcomes of patients with geriatric hip fractures after surgery.

**Level of Evidence:**

Level II, therapeutic study. See the guidelines for authors for a complete description of levels of evidence.

## Introduction

In modern practice, postoperative care of patients with geriatric hip fractures is critically dependent on early mobilization [5, 25, 35, 44]. Early mobilization reduces complications such as thromboembolism, bedsores, and pulmonary complications. However, postoperative pain often hinders early mobilization and weightbearing exercises [1, 40]. There are many factors complicating pain management in elderly patients that may not be adequately addressed by providing analgesics as required, which has been the standard approach to postoperative pain management at our center.

Pain management begins with assessment. However, the assessment of pain in elderly patients can be difficult. Elderly patients may be less inclined to report their pain for various reasons, including a fear-avoidance mentality, disinclination to trouble their caregivers, the stoic nature of their personalities, cultural influence, and others [2, 6, 7]. Moreover, a large number of patients with geriatric hip fractures have limited cognitive function, further intensifying the difficulty of assessment.

Underreporting of pain by patients for any of the reasons described above can lead to the undertreatment of pain [7, 8, 24]. Reports also indicate that nurses may be inclined to undertreat patients with pain for various reasons, including concerns about the side effects of analgesics and a “play it safe” mentality, among others [42, 54]. In addition, pain assessments typically are done in patients who are resting, which may not reflect the level of pain that patients will experience when they begin to ambulate during therapy.

We postulate that inadequate pain control can negatively affect the rate of functional recovery in the rehabilitation process after hip fracture surgery. Accordingly, we compared an approach using scheduled analgesic dosing with as-needed analgesic dosing in patients after hip fracture surgery, to compare these approaches in terms of (1) resting and dynamic pain intensity, (2) postoperative patient mobility, and (3) functional end points.

## Patients and Methods

We prospectively reviewed the efficacy of two analgesic regimens on rehabilitation outcomes of patients with hip fractures in an orthopaedic rehabilitation center during a 2-year period (2010–2012). We included 480 patients with (1) age of 65 years or older; (2) femoral neck, trochanteric, or subtrochanteric fractures; and (3) surgery with internal fixation or arthroplasty performed. Exclusion criteria included (1) patients with intolerance to the drug regimen; (2) patients taking other medications that contraindicated or interacted with the drug regimen; and (3) patients who refused to participate in the study. Eighty patients were excluded from the study according to our prespecified exclusion criteria. This left 400 patients who were included in the study. Two separate regimens were implemented in these two years. The intervention group, consisting of 200 consecutive patients admitted for hip fracture surgery between February and August 2010, received a scheduled analgesic protocol, consisting of tramadol 50 mg and paracetamol 500 mg three times a day for 3 weeks and then the same drugs three times a day as needed (pro re nata [PRN]) thereafter. The control group, consisting of the next 200 patients admitted to our center for hip fracture surgery, between September 2011 and March 2012, received analgesics on a PRN basis, including tramadol 50 mg and paracetamol 500 mg up to six times a day on request by the patient. The groups were comparable in terms of baseline characteristics (Table 1). Among the patients excluded from the study, 8% in the control group had gastrointestinal upset develop with tramadol, 6% had contraindications or interactions with tramadol, and 2% refused to participate in the study. In the intervention group, 9% had gastrointestinal upset develop with tramadol, 7% had contraindications or interactions with tramadol, and 2% refused to participate in the study. Tramadol [15, 61] is a centrally acting synthetic opioid analgesic. Paracetamol [14, 60] is a nonopiate, nonsalicylate analgesic. We used a combination of both analgesics with different pharmacologic mechanisms to improve the efficacy of pain control and to minimize side effects [11, 18, 20, 41]. We obtained approval from our Institutional Research Ethical Board and Clinical Trial Board. All patients gave written consent to participate in the study. For patients with impaired cognition or communication, their healthcare power of attorney gave consent for them.

**Table 1:** Demographic and clinical characteristics of the patients

Characteristics	Control group (N = 200)	Intervention group (N = 200)	p Value*
Age (years)			
Mean (SD)	84.02 (6.40)	82.84 (6.74)	0.073
Median (range)	84.00 (66–100)	83.50 (66–99)	
Sex (number of patients)			
Male	62 (31.0%)	70 (35.0%)	0.457
Female	138 (69.0%)	130 (65.0%)	
Fracture (number of patients)			
Neck of femur	93 (46.5%)	84 (42.0%)	0.108
Trochanter	99 (49.5%)	97 (48.5%)	
Subtrochanter	8 (4.0%)	19 (9.5%)	
Surgery (number of patients)			
Closed reduction internal fixation	129 (64.5%)	122 (61.0%)	0.535
Arthroplasty	71 (35.5%)	78 (39.0%)	
Abbreviated mental test (number of patients)			
Abbreviated Mental Test 6–10	116 (58.0%)	126 (63.0%)	0.357
Abbreviated Mental Test 0–5	84 (42.0%)	74 (37.0%)	
Comorbidity (number of patients)			
Without CVA/parkinsonism	166 (83.0%)	168 (84.0%)	0.893
With CVA/parkinsonism	34 (17.0%)	32 (16.0%)	
Ambulation (premorbid) (number of patients)			
Unaided	70 (35.0%)	72 (36.0%)	0.761
Stick	103 (51.5%)	94 (47.0%)	
Quadripod	7 (3.5%)	12 (6.0%)	
Frame	18 (9.0%)	20 (10.0%)	
Wheelchair	2 (1.0%)	2 (1.0%)	
Modified functional ambulation categories (premorbid) (number of patients)			
Sitter	3 (1.5%)	2 (1.0%)	0.186
Dependent walker	6 (3.0%)	6 (3.0%)	
Assisted walker	16 (8.0%)	13 (6.5%)	
Supervised walker	8 (4.0%)	22 (11.0%)	
Indoor walker	53 (26.5%)	53 (26.5%)	
Outdoor walker	114 (57.0%)	104 (52.0%)	
Pressure sore (admission) (number of patients)			
No sores	157 (78.5%)	156 (78.0%)	1.000
Has sores	43 (21.5%)	44 (22.0%)	
Urology (admission) (number of patients)			
Self-voiding	163 (81.5%)	168 (84.0%)	0.597
Indwelling catheter	37 (18.5%)	32 (16.0%)	
Residence (premorbid) (number of patients)			
Home	162 (81.0%)	158 (79.0%)	0.708
Elderly home	38 (19.0%)	42 (21.0%)	
Social assistance (premorbid) (number of patients)			
Old age allowance	133 (66.5%)	128 (64.0%)	0.675
Disability allowance/high disability allowance/ comprehensive social security assistance	67 (33.5%)	72 (36.0%)	
Numerical Rating Scale at rest (admission) (points)			
Mean (SD)	3.76 (2.41)	3.62 (1.61)	0.480
Median (range)	4.00 (0–10)	3.00 (0–9)	
Numerical Rating Scale during activity (admission) (points)			
Mean (SD)	4.32 (2.39)	4.26 (2.06)	0.805
Median (range)	4.50 (0–10)	4.00 (0–10)	
Elderly Mobility Scale (admission) (points)			
Mean (SD)	4.46 (1.75)	4.14 (1.97)	0.092
Median (range)	4.00 (0–10)	4.00 (0–11)	
Functional Independence Measure™ (admission) (points)			
Mean (SD)	62.14 (12.45)	64.24 (15.39)	0.135
Median (range)	64.00 (20–86)	64.00 (22–94)	

* Chi-square for proportions, t-test for continuous variables; CVA = cerebrovascular accident.

We used Altman’s nomogram [46] to estimate the sample size of subjects necessary to have a 90% power to detect a 10-point difference in the Functional Independence Measure™ [22] between the two groups at the 5% level of significance. The tool had adequate consistency in various elderly populations and concurrent validity with other functional measures [13, 21, 68]. We assumed that the standard deviation of the Functional Independence Measure™ was approximately 15. We used the nomogram to estimate the required sample sizes of two groups, with δ = 10 and σ = 15. The standardized difference equaled δ/σ = 10/15 = 0.67. The line connecting a standardized difference of 0.67 and a power of 90% cut the sample size axis at approximately 98. Therefore, approximately 49 subjects were required for each group.

We used several instruments to assess our patients. We used the modified Abbreviated Mental Test [27] to assess patients’ cognition. The tool was validated by Sarasqueta et al. [49] with a 91.5% sensitivity and 82.4% specificity. We used a 0 to 10 Numerical Rating Scale [7, 9, 36] for patients who were able to express the intensity of their current pain on a scale of 0 (no pain) to 10 (worst possible pain). We used this tool because it was simple and could be verbally delivered. Evidence has supported the reliability and validity of the tool across many populations including the Chinese population [17, 26, 33, 63]. Pain at rest was measured weekly by nurses in the ward. Pain during activity was measured weekly by physiotherapists during exercises [19]. Based on Krebs et al. [30], a Numerical Rating Scale of 1 to 3 was defined as minimal pain, 4 to 6 as moderate pain and 7 to 10 as severe pain. We determined that patients had adequate control of their pain when the pain was between 0 and 3. Forty percent of patients had cognitive impairment. When they were unable to produce a Numerical Rating Scale, a Wong-Baker FACES^®^ Pain Rating Scale [31, 50, 65] was used. The tool had six faces with a score from 0 to 10, starting with a “no hurt” face on the left to a “hurts worst” face on the right. This tool has adequate consistency in various pain populations and concurrent validity with other pain instruments [38]. Although the tool was developed for use in children, it is validated to be useful for elderly with disparities in cognition, communication, and literacy [4, 31, 43, 57]. Guidelines and in-service training were provided to staff before our study. Interviews using an information brochure were provided to all participants before the study. Patients with sound cognition were educated on (1) how to give ratings on pain intensity, and (2) the regimen, rationale, effects, and adverse effects of analgesics. For patients with impaired cognition or communication, staff guided patients to point out the severity of their pain using a card with enlarged face images indicating the Wong-Baker FACES^®^ Pain Rating Scale [65], or gave ratings according to patients’ facial expressions when they could not use a face scale. In this situation, analgesics were administered as needed mainly based on nursing judgment.

We used the Elderly Mobility Scale [10, 32, 52, 69] to assess the progress of patients’ mobility. The scale assessed seven motor function items to indicate mobility capacity and basic activities of daily living. Kuys and Brauer [32] examined the concurrent validity of the Elderly Mobility Scale by correlating it with the Barthel Index and Functional Independence Measure™ scores for a group of elderly patients. Spearman’s rho was 0.962 with the Barthel Index and 0.948 with the Functional Independence Measure. The scale was measured weekly by physiotherapists. We used the Functional Independence Measure™ to assess the functional outcomes of patients. The tool expresses the level of human assistance required for a person to perform daily activities. It consists of 13 motor and five cognitive items on a scale of 1 to 7 based on the level of independence for each functional item [23, 28, 29, 68]. The scale was measured by occupational therapists on admission and on discharge. We used the Modified Functional Ambulation Category scale [64] to categorize the ambulatory status of patients. The tool was a modified version of the Functional Ambulation Category which took into account the use of walking aids. The tool was divided into seven categories from I to VII (I = layer; II = sitter; III = dependent walker; IV = assisted walker; V = supervised walker; VI = indoor walker; VII = outdoor walker). Mehrholz et al. [37] examined the validity of the tool and the results indicated that it had excellent reliability, good concurrent and predictive validity, and good responsiveness in patients. The tool was measured by physiotherapists before admission and on discharge.

The trend of analgesic consumption in the control and intervention groups were compared (Table 2). Of the 200 patients in the control group, 173 (87%) patients took analgesics, of which 131 (66%) took tramadol and paracetamol and 42 (21%) took paracetamol only, based on their preference. Twenty-seven (13%) patients did not take any analgesics during the entire hospitalization. Even during the first week, not all patients took analgesics, and, as the weeks passed, the number of patients who consumed analgesics and the frequency with which the analgesics were taken gradually decreased. In comparison, the intervention group received a larger total dose of analgesics than the control group (tramadol doses, 63 versus 27; paracetamol doses, 63 versus 29; p < 0.001). The intervention group also took more days to take analgesics than the control group (tramadol days, 21 versus 11; paracetamol days, 21 versus 13; p < 0.001). Constipation requiring administration of bisacodyl suppositories was observed. Ten milligrams of bisacodyl suppository daily prn was prescribed for each patient in both groups. One hundred thirty-one patients (66%) in the intervention group required bowel management with a mean amount of bisacodyl suppository of 2.6 ± 1.4 received. One hundred eighteen patients (59%) in the control group required bowel management with a mean amount of bisacodyl suppository of 4.7 ± 4.1 received. Other than postoperative pain, some patients also experienced other types of pain, mainly lower back pain, gout, or osteoarthritis, and required NSAIDs. There were no differences between the duration and quantity of these drugs taken between the two groups during hospitalization. Patients were discharged based on several criteria, including achievement of medical stability and pain control at the Numerical Rating Scale of 0 to 3, attainment of a plateau in training progress, availability of appropriate placement, and a suitable caregiver as indicated.

**Table 2:** Comparison of drug consumption for the two groups

Variable	Control group		Intervention group		t Value	p Value
	Number of patients	Mean (SD)	Number of patients	Mean (SD)		
Amount of tramadol taken (doses)						
Week 1	126 (63.0%)	13.06 (8.03)	200 (100%)	21.00 (0.00)	−11.095	< 0.001
Week 2	99 (49.5%)	11.79 (8.09)	200 (100%)	21.00 (0.00)	−11.328	< 0.001
Week 3	57 (28.5%)	12.49 (8.50)	200 (100%)	20.85 (1.43)	−7.392	< 0.001
3 weeks in total	131 (65.5%)	26.91 (22.69)	200 (100%)	62.85 (1.43)	−18.103	< 0.001
Amount of paracetamol taken (doses)						
Week 1	169 (84.5%)	13.33 (7.89)	200 (100%)	21.00 (0.00)	−12.637	< 0.001
Week 2	143 (71.5%)	12.05 (7.54)	200 (100%)	21.00 (0.00)	−14.196	< 0.001
Week 3	97 (48.5%)	11.38 (7.76)	200 (100%)	20.85 (1.43)	−11.921	< 0.001
3 weeks in total	173 (86.5%)	29.36 (21.77)	200 (100%)	62.85 (1.43)	−20.196	< 0.001
Duration of tramadol taken (days)						
Week 1	126 (63.0%)	5.51 (2.09)	200 (100%)	7.00 (0.00)	−8.003	< 0.001
Week 2	99 (49.5%)	4.99 (2.34)	200 (100%)	7.00 (0.00)	−8.560	< 0.001
Week 3	57 (28.5%)	5.21 (2.35)	200 (100%)	6.95 (0.48)	−5.554	< 0.001
3 weeks in total	131 (65.5%)	11.34 (7.20)	200 (100%)	20.95 (0.48)	−15.261	< 0.001
Duration of paracetamol taken (days)						
Week 1	169 (84.5%)	5.71 (1.95)	200 (100%)	7.00 (0.00)	−8.573	< 0.001
Week 2	143 (71.5%)	5.43 (2.16)	200 (100%)	7.00 (0.00)	−8.695	< 0.001
Week 3	97 (48.5%)	5.20 (2.31)	200 (100%)	6.95 (0.48)	−7.395	< 0.001
3 weeks in total	173 (86.5%)	12.98 (7.05)	200 (100%)	20.95 (0.48)	−14.843	< 0.001
Consumption of laxative (bisacodyl suppositories)	131 (65.5%)	2.60 (1.43)	118 (59.0%)	4.65 (4.11)	0.216	< 0.001
Consumption of other analgesics during hospitalization (days)						
Naprosyn 250 mg three times a day prn	6 (3.0%)	31.50 (18.43)	11(5.5%)	31.00 (8.80)	0.077	0.940
Voltaren 100 mg daily prn	8 (4.0%)	30.00 (12.67)	9 (4.5%)	30.33 (6.38)	−0.070	0.945
Consumption of other analgesics during hospitalization (doses)						
Naprosyn 250 mg three times a day prn	6 (3.0%)	63.00 (36.85)	11(5.5%)	62.00 (17.59)	0.077	0.940
Voltaren 100 mg daily prn	8 (4.0%)	30.00 (12.67)	9 (4.5%)	30.33 (6.38)	−0.070	0.945

Prn = as needed.

We performed all statistical analyses using SPSS^®^ software (Version 17.0; SPSS Inc, Chicago, IL, USA). We used chi-square tests to compare categorical variables. We used paired t-tests to compare the changes of continuous variables within groups and independent t-tests to compare the changes of continuous variables between groups. We used the Pearson correlation test to perform simple correlation analyses. Statistical significance was conferred by a two-tailed p value of 0.05 or less. The interrater reliabilities for the Numerical Rating Scale were 0.874 at rest and 0.882 during activity. The interrater reliabilities for the Wong-Baker FACES^®^ Pain Rating Scale were 0.860 at rest and 0.870 during activity. The interrater reliability for the Elderly Mobility Scale was 0.913. The interrater reliability for the Functional Independence Measure™ was 0.927, and the interrater reliability for the Modified Functional Ambulation Category was 0.901.

## Results

Patients experienced a greater level of dynamic pain than resting pain on admission. As expected, analgesic consumption was lower in the control group (tramadol doses, 27 versus 63; paracetamol doses, 29 versus 63) (Table 2). Despite the large difference in the amount of analgesics consumed, each group showed improvement in resting and dynamic pain intensity (p < 0.001) (Table 3). In addition, there was a positive correlation between functional outcomes and analgesic consumption in the control group (Table 4). However, there was no difference between groups in terms of postoperative pain at different times (Table 5). Patients presented with mild to moderate resting pain in the control and intervention groups on admission (3.76 ± 2.41 versus 3.62 ± 1.61). The patients achieved gradual pain improvement at Weeks 1 and 2 and had mild resting pain on discharge (1.62 ± 1.32 versus 1.45 ± 1.29). During activity, patients had moderate dynamic pain in the control and intervention groups on admission (4.32 ± 2.39 versus 4.26 ± 2.06). The patients experienced gradual pain improvement at Weeks 1 and 2 and had mild dynamic pain on discharge (1.91 ± 1.62 versus 1.72 ± 1.48).

**Table 3:** Descriptive statistics for NRS, EMS, and FIM™ for the two groups

Variable	Control group (n = 200)				Intervention group (n = 200)			
	Mean (SD)		t Value	p Value	Mean (SD)		t Value	p Value
	Admission	Discharge			Admission	Discharge		
NRS at rest (points)	3.76 (2.41)	1.62 (1.32)	14.990	<0.001	3.62 (1.61)	1.45 (1.29)	19.059	< 0.001
NRS during activity (points)	4.32 (2.39)	1.91 (1.62)	18.352	<0.001	4.26 (2.06)	1.72 (1.48)	17.635	< 0.001
EMS (points)	4.46 (1.75)	8.30 (3.03)	−24.436	<0.001	4.14 (1.97)	11.00 (2.94)	−46.611	< 0.001
FIM™ (points)	62.14 (12.45)	79.15 (18.31)	−27.664	<0.001	64.24 (15.39)	88.42 (17.43)	−39.306	< 0.001

NRS = Numerical Rating Scale; EMS = Elderly Mobility Scale; FIM™ = Functional Independence Measure.

**Table 4:** Correlation between EMS, FIM™, and drug consumption of 200 patients in the control group

Variable	EMS on discharge	FIM on discharge	Total doses of tramadol intake	Total doses of paracetamol intake	Total days of tramadol intake	Total days of paracetamol intake
EMS on discharge	–					
FIM on discharge	0.756*	–				
Total doses of tramadol intake	0.176^†^	0.221*	–			
Total doses of paracetamol intake	0.172^†^	0.230*	0.796*	–		
Total days of tramadol intake	0.169^†^	0.214*	0.927*	0.695*	–	
Total days of paracetamol intake	0.144^†^	0.193*	0.642*	0.886*	0.671*	–

* p < 0.01, Pearson correlation; ^†^p < 0.05, Pearson correlation; EMS = Elderly Mobility Scale; FIM™ = Functional Independence Measure.

**Table 5:** Comparison of NRS, EMS, FIM™, ambulation and MFAC for the two groups

Variable	Control group (n = 200)	Intervention group (n = 200)	t or χ^2^ Value*	p Value
NRS at rest (points)^†^				
Admission	3.76 (2.41)	3.62 (1.61)	0.707	0.480
Week 1	2.90 (1.99)	2.90 (1.46)	0.029	0.977
Week 2	2.39 (1.83)	2.34 (1.54)	0.278	0.781
Week 3	1.95 (1.64)	1.93 (1.44)	0.154	0.878
Discharge	1.62 (1.32)	1.45 (1.29)	1.367	0.172
NRS at rest (mean difference) (points)^†^				
Between Week 1 and admission	−0.86 (1.22)	−0.72 (1.21)	−1.155	0.249
Between Week 2 and Week 1	−0.52 (0.85)	−0.55 (0.92)	0.309	0.758
Between Week 3 and Week 2	−0.57 (0.83)	−0.46 (0.89)	−1.037	0.300
Between discharge and admission	−2.14 (2.04)	−2.17 (1.64)	0.165	0.869
NRS during activity (points)^†^				
Admission	4.32 (2.39)	4.26 (2.06)	0.247	0.805
Week 1	3.41 (2.03)	3.51 (2.06)	−0.490	0.624
Week 2	2.73 (1.81)	2.96 (2.02)	−1.231	0.219
Week 3	2.32 (1.64)	2.40 (1.59)	−0.640	0.523
Discharge	1.91 (1.62)	1.72 (1.48)	1.480	0.140
NRS during activity (mean difference) (points)^†^				
Between Week 1 and admission	−0.91 (1.04)	−0.76 (1.48)	−1.213	0.226
Between Week 2 and Week 1	−0.70 (0.89)	−0.54 (1.12)	−1.509	0.132
Between Week 3 and Week 2	−0.55 (0.85)	−0.66 (1.05)	0.856	0.392
Between discharge and admission	−2.41 (2.03)	−2.54 (1.99)	0.667	0.505
EMS (points)^†^				
Admission	4.46 (1.75)	4.14 (1.97)	1.688	0.092
Week 1	5.86 (2.20)	5.82 (2.22)	0.158	0.874
Week 2	7.17 (2.61)	8.15 (2.66)	−3.733	< 0.001
Week 3	8.14 (2.66)	10.34 (3.06)	−7.236	< 0.001
Discharge	8.30 (3.03)	11.00 (2.94)	−9.076	< 0.001
EMS (mean difference) (points)^†^				
Between Week 1 and admission	1.40 (1.03)	1.68 (0.88)	−2.922	0.004
Between Week 2 and Week 1	1.30 (0.92)	2.32 (1.12)	−10.042	< 0.001
Between Week 3 and Week 2	0.86 (0.83)	2.27 (0.99)	−14.449	< 0.001
Between discharge and admission	3.84 (2.22)	6.86 (2.08)	−14.045	< 0.001
FIM™ (points)^†^				
Admission	62.14 (12.45)	64.24 (15.39)	−1.496	0.135
Discharge	79.14 (18.31)	88.42 (17.43)	−5.192	< 0.001
FIM™ (mean difference) between discharge and admission (points)^†^	17.00 (8.69)	24.19 (8.70)	−8.262	< 0.001
Ambulation (discharge) (number of patients)				
Unaided	2 (1.0%)	3 (1.5%)	40.241	< 0.001
Stick	6 (3.0%)	27 (13.5%)		
Quadripod	33 (16.5%)	47 (23.5%)		
Frame	127 (63.5%)	120 (60.0%)		
Wheelchair	32 (16.0%)	3 (1.5%)		
MFAC (discharge) (number of patients)				
Sitter	32 (16.0%)	3 (1.5%)	43.672	< 0.001
Dependent walker	42 (21.0%)	26 (13.0%)		
Assisted walker	86 (43.0%)	104 (52.0%)		
Supervised walker	36 (18.0%)	44 (22.0%)		
Indoor walker	2 (1.0%)	11 (5.5%)		
Outdoor walker	2 (1.0%)	12 (6.0%)		

* χ^2^ values given for last two variables, ambulation and MFAC at discharge; ^†^ values are expressed as mean, with SD in parentheses; NRS = Numerical Rating Scale; EMS = Elderly Mobility Scale; FIM™ = Functional Independence Measure; MFAC = Modified Functional Ambulation Category.

Although similar pain intensities were perceived by patients in both groups, the intervention group had a better ambulatory status at discharge (p < 0.001) (Table 5). On discharge, more patients in the intervention group were able to walk with different types of aids (unaided, 3 versus 2; stick, 27 versus 6; quadripod, 47 versus 33; frame, 120 versus 127), and fewer patients required the use of a wheelchair (3 versus 32). The outcomes also were evident with the Modified Functional Ambulation Category. The total percentage of indoor or outdoor walkers was 11.5% in the intervention group and 2% in the control group. The total percentage of supervised or assisted walkers was 74% in the intervention group and 61% in the control group. The total percentage of dependent walkers was 13% in the intervention group and 21% in the control group. The total percentage of sitters was 1.5% in the intervention group and 16% in the control group (p < 0.001).

Apart from enhanced ambulation outcomes, the intervention group achieved better mobility performance on discharge (Elderly Mobility Scale, 11 versus 8) (Table 5). With scheduled and adequate dosing of analgesics, patients showed steady mobility improvement at different times (p < 0.001 at Week 2, Week 3, and at discharge). The improvement also was seen in the mean difference after each week of training (p = 0.004 after 1 week of training, p < 0.001 after 2 weeks and 3 weeks of training). In addition to mobility enhancement, the intervention group showed higher functional performance on discharge (Functional Independence Measure™, 88 versus 79; p < 0.001). The mean Functional Independence Measure™ difference between discharge and admission for both groups was 24 and 17 respectively (p < 0.001).

The rate of complications occurring during the course of rehabilitation was similar. There were no fall incidents during hospitalization. Length of stay was longer in the intervention group. According to clinical experience, this discrepancy was attributable mainly to social factors, including time consumption in considering and arranging for placement and caregivers on discharge (Table 6). There was no difference in outcomes at 6 months after discharge between groups (Table 7).

**Table 6:** Comparison of outcomes for the two groups

Variable	Control group (n = 200)	Intervention group (n = 200)	χ^2^ Value	p Value
Complications during rehabilitation (number of patients)				
Chest infection	20 (10.0%)	14 (7.0%)	1.157	0.370
Urinary tract infection	43 (21.5%)	40 (20.0%)	0.137	0.805
Wound infection	17 (8.5%)	15 (7.5%)	0.136	0.854
Retention of urine with Foley catheterization	22 (11.0%)	25 (12.5%)	0.217	0.641
Sepsis	33 (16.5%)	28 (14.0%)	0.484	0.578
Pressure sore	24 (12.0%)	23 (11.5%)	0.024	1.000
Placement at discharge				
Home	126 (63.0%)	121 (60.5%)	0.265	0.681
Elderly home	74 (37.0%)	79 (39.5%)		
Length of stay (days)				
Mean (SD)	25.02 (12.03)	28.64 (8.95)	−3.425	< 0.001
Median (range)	23.00 (7–95)	28.00 (7–47)

**Table 7:** Comparison of outcomes within 6 months after discharge to community

Variable	Number of patients		χ^2^ Value	p Value
	Control group (n = 200)	Intervention group (n = 200)		
Emergency attendance headcount	60 (30.0%)	56 (28.0%)	0.194	0.741
Emergency attendance episodes				
1	43 (21.5%)	39 (19.5%)	0.251	0.882
> 1	17 (8.5%)	17 (8.5%)		
Unplanned readmission within 6 months				
Medical problems	31 (15.5%)	28 (14.0%)	0.610	0.894
Surgical problems	5 (2.5%)	5 (2.5%)	1.111	0.574
Pneumonia	14 (7.0%)	12 (6.0%)	1.211	0.750
Contusion of hip	3 (1.5%)	6 (3.0%)	1.523	0.467
Fractures	5 (2.5%)	3 (1.5%)	0.510	0.724
Implant complication	1 (0.5%)	3 (1.5%)	1.343	0.511
Fall	7 (3.5%)	9 (4.5%)	0.260	0.800
Mortality headcount	12 (6.0%)	11 (5.5%)	0.046	1.000
Causes of mortality				
Cardiac problem	1 (0.5%)	0 (0.0%)		
Sepsis	2 (1.0%)	0 (0.0%)		
Stroke	0 (0.0%)	1 (0.5%)	6.061	0.416
Pneumonia	8 (4.0%)	9 (4.5%)		
Urinary tract infection	1 (0.5%)	0 (0.0%)		
Cancer	0 (0.0%)	1 (0.5%)		

## Discussion

The importance of postoperative pain control in patients with geriatric hip fractures is well recognized [2, 5, 7, 9, 51], and in this study we sought to compare two different approaches to analgesia after hip fracture surgery. We compared scheduled oral analgesic administration with administration of pain medications as needed; our premise was that we may have undertreated pain in our patients before, and that increasing analgesic dosage may improve pain control and result in better response to rehabilitation. We found that using scheduled analgesics did not result in improved pain scores, but did appear to result in faster and more complete functional rehabilitation outcomes.

This study has numerous limitations. First, our major limitation was that the study was not a prospective randomized study. In addition, our large setting made it impossible for us to limit the number of assessors to a small number of staff to minimize interrater differences. However with on-the-job training, our interobserver reliability data suggested that this limitation was not severe. In elderly patients, one must monitor carefully for drug complications and drug interactions; this close monitoring resulted in a dropout rate of approximately 8% in the control group and 9% in the intervention group owing to nausea, and 6% in the control group and 7% in the intervention owing to contraindications or interactions with other medications. Forty-two percent of our control group and 37% of our intervention group had impaired cognition which could lead to an inadequate response to our Numerical Rating Scale assessments [7]. In this situation we used the Wong-Baker FACES^®^ Pain Rating Scale [65] to improve the assessment. In our locality, length of stay relied much more on social background and discharge problem solving than physical conditions. Therefore, it was difficult to evaluate our results regarding these outcome data.

Finally, we did not compare results according to several parameters including (1) types of fractures and surgeries, (2) comorbidity, and (3) postoperative complications, especially delirium which would have different responses on pain. In addition, 13% of our patients in the control group did not take any analgesic medication. The percentage of impaired cognition in this group and the impact on functional outcomes could be explored. A quantitative measurement of walking ability would strengthen the study.

The answer to our question whether scheduled analgesic dosing after hip fracture surgery would decrease patients’ pain is somewhat counterintuitive and deserves further discussion. Similar to the study by Feldt and Oh, movement pain was substantially greater than resting pain in the groups [16]. Despite regular and greater analgesic doses administered, pain scores were not different between the groups at rest and during movement. Subjective pain scores generally are accepted to be satisfactory as a measure for pain in the elderly, even for patients with mild cognitive problems [33]. However, some authors have suggested the necessity to use multidimensional assessment methods for accurate pain assessment good enough for pain management in the elderly [6–8]. The National Guidelines [9] described that pain is a complex and personal experience. It is affected by physiologic, psychologic, social, and cultural influences. The pain experience can be described at different levels. The sensory dimension describes the location, quality, and severity of the pain sensation. The affective dimension describes the emotional responses to pain. The impact dimension describes the effects of pain on the person’s functioning. In the sensory dimension, we used a numerical rating scale [7] to measure pain intensity. Von Baeyer [58] and Narayan [42] reported that individual patients would perceive and tolerate pain differently. Therefore, a subjective score of severe pain expressed by a patient may not be as severe as a score of moderate pain expressed by another patient. To have a better view of pain experiences, numerous studies had been conducted to identify some biobehavioral markers of pain experience, such as fear and anxiety on radiant heat pain thresholds [48], heart rate and heat stimuli of different intensities [34], blood pressure and chronic pelvic pain syndrome [67], and sleep quality and acute postoperative pain after hip and knee arthroplasties [66]. Our pain scores communicated limited information regarding impact of pain experiences in our patients. From our findings, it was unclear whether our elderly patients experienced less pain or reported less pain. We have strengthened our belief regarding the dubious reliability of subjective pain scores in elderly patients as a measure to guide pain management. In light of this, we used other strategies to study pain and pain treatment, predominately focusing on the observation of behaviors in terms of walking and functioning abilities.

The answer to our question regarding whether scheduled analgesic dosing after hip fracture surgery would enhance patient mobility was encouraging. Studies in postoperative samples have shown that early ambulation was important for recovery [53, 56]. Rehabilitation started with walking exercises, and pain could be provoked during movement in addition to postoperative pain. It also has been reported that pain was associated with delayed ambulation and long-term functional impairment [40], impaired compliance to physiotherapy [1], poor instrumental and social functioning [62], and mortality [56]. Good pain control can enhance patient participation and performance during rehabilitation exercises [2, 16, 39, 62]. Benefits afforded by our time-scheduled analgesia were extended to mobilization status and ambulatory category. The majority of our patients started with a walking frame or a rollator for training. More patients in our intervention group had a faster response to training and transit to cane or quadripod walker (37.0% versus 19.5%) on discharge. This indicated that the patients had improved stability and required less dependence on a frame. Our findings were further evidenced by the Modified Functional Ambulation Category. Fewer patients were dependent walkers or sitters in the intervention group (14.5% versus 37.0%), which indicated that there was less postfracture severe disability in this group.

The answer to our third question, whether scheduled analgesic dosing after hip fracture surgery would improve functional end points also was encouraging. The efficacy of our pain regimen was not reflected through a subjective pain score but was shown by the more objective Elderly Mobility Scale and Functional Independence Measure™ end points. In a study of functional outcomes, Arinzon et al. [3] found that postoperative pain was an independent predictor of the Functional Independence Measure™ on discharge and with every increase in one point of VAS on admission greater than 4 points [63], the Functional Independence Measure™ on discharge decreased by 8.77. In a similar study conducted by Zabari et al. [70], the control group was treated with a single analgesic including acetaminophen, whereas the study group was treated with acetaminophen with additional tramadol or dipyrone to titrate for pain. They found that patients had higher Functional Independence Measure™ scores between admission and discharge in the study group (11.07 ± 7.9 versus 8.4 ± 7 .3; p < 0.03). Numerous studies showed that a time-scheduled pattern was better than a pain-contingent pattern (PRN) in postoperative pain management. The former with adequate dosing could provide stable therapeutic blood levels and a continuous relieving effect for acute pain [12, 45, 47, 55, 59]. Our time-scheduled analgesic was consistent with this principle and its efficacy was observed by better functional end points.

We found improved ambulatory status and functional outcomes in our patients after surgery for geriatric hip fractures by using a scheduled approach to analgesic delivery, which also effectively increased the analgesic dosage for patients during the first few weeks after fracture. However, not all pain in this patient population is from the surgery. Pain in these patients can come from several sources, including degenerative conditions of the hips or knees, pressure sores, concomitant injuries, or other medical conditions. In addition, analgesic medication is only one of the multiple methods for treatment for pain. In light of that, pain management should be individualized after clinical assessment of each patient.
